# XGBoost-based model for predicting five-year survival in gastric cancer using clinical indicators

**DOI:** 10.1038/s41598-026-50043-x

**Published:** 2026-04-25

**Authors:** Yujiao Zhang, Xuemeng Zhou, Peixian Li, Chunfeng Li, Lixia Ke

**Affiliations:** 1 Department of Oncology, Beidahuang Industry Group General Hospital, Harbin, China; 2 Department of Gastrointestinal Surgery, Harbin Medical University Cancer Hospital, Harbin, China

**Keywords:** gastric cancer, prognosis, machine learning, clinical indicators, model, Biomarkers, Cancer, Computational biology and bioinformatics, Gastroenterology, Oncology

## Abstract

Accurate diagnosis and prognostic assessment of gastric cancer are critical for improving patient outcomes. The application of advanced machine learning methods, particularly the XGBoost algorithm, offers a promising approach for enhancing prognostic evaluations. In this study, data from 2,270 patients with gastric cancer were analysed to develop a predictive model for prognosis using the XGBoost algorithm and 20 key clinical features. Comprehensive data collection, preprocessing, and feature selection were conducted to ensure robust model construction and validation. The model demonstrated strong predictive performance in the test cohort, achieving an area under the curve (AUC) of 0.855, and it effectively differentiated patients at high-risk from those at low-risk. Feature importance analysis revealed that pTNM stage and CA125 level were the most influential prognostic factors. This study successfully implemented a machine learning-based model integrating the XGBoost algorithm and critical clinical indicators to predict the five-year survival rate of patients with gastric cancer. The findings highlight the potential of such approaches in supporting personalised treatment strategies and advancing cancer prognosis assessment methodologies.

**Supplementary Information:**

The online version contains supplementary material available at 10.1038/s41598-026-50043-x.

## Introduction

Gastric cancer is a major public health issue worldwide and is among the leading causes of cancer-related deaths^1,2^. The prognosis is often poor because of late-stage diagnosis and limited treatment effectiveness. Despite advancements in treatment methods and strategies over the past few decades^3–5^, improvements in the survival period for patients with gastric cancer remain limited, highlighting the importance of early diagnosis and precise prognostic assessment. In recent years, with the development of statistics and bioinformatics, the use of machine learning algorithms to predict disease prognosis has become possible^6,7^. Research in various medical fields, such as oncology^8^ and cardiology^9^, has demonstrated the potential of the XGBoost algorithm for enhancing diagnostic accuracy and prognostic prediction.

Recent studies on lung adenocarcinoma have further confirmed the great potential of machine learning in cancer prognosis prediction and treatment guidance. For example, studies have identified disulfidptosis-related molecular subtypes and prognostic signatures and have constructed nomograms integrating clinical variables to improve survival prediction accuracy. Furthermore, these studies have revealed the regulatory role of related genes in the tumour microenvironment and treatment response. Machine learning-based analyses of single-cell transcriptomes have clarified the correlation between EGFR mutations and tumour immune microenvironment subtypes, providing new insights for precise immunotherapy. In addition, metabolic reprogramming-related signatures constructed via machine learning have been proven to effectively predict immunotherapy efficacy and identify key therapeutic targets. Collectively, these findings indicate that integrating machine learning with molecular, immune and clinical features is an effective way to improve the precision of cancer prognostic prediction^10–12^.

Clinical features such as pTNM staging^13^, biomarkers (such as CA125)^14^, patient age^15^, the presence of vascular tumour thrombi^16^, and tumour size^17^ have been proven to be closely related to patient survival in studies on the prognostic prediction of gastric cancer. The rational and comprehensive application of these clinical features is particularly crucial for constructing a high-precision prognostic model. Currently, efficiently integrating these variables to guide clinical practice remains a significant challenge. In this study, the value of combining the XGBoost algorithm with clinical features for predicting the five-year survival rate of patients with gastric cancer is explored, with the goal of providing a scientific basis for the personalised treatment and management of these patients.

## Patients and methods

### Patient selection

In this study, 2270 patients with gastric cancer were recruited from January 2014 to December 2016 at the Harbin Medical University Cancer Hospital.

The inclusion criteria were as follows: (1) patients who underwent a histological biopsy and had postoperative pathology-confirmed gastric cancer; (2) patients who underwent curative gastric radical R0 resection combined with standard D2 or D2 + lymph node dissection; (3) patients for whom complete clinicopathological information was available or for whom the proportion of missing data did not exceed 30%, guaranteeing overall data integrity and reliability; and (4) patients with complete follow-up data (all lost-to-follow-up cases were excluded from the study and not included in the final analysis).

The exclusion criteria were as follows: (1) patients who had received preoperative neoadjuvant radiotherapy, chemotherapy, or combined antitumour therapy; (2) patients who died within 30 days after surgery, to eliminate the confounding effects of acute postoperative complications; (3) patients with other concurrent primary malignant tumours; and (4) patients with severe hepatic or renal insufficiency.

### Data collection

Patient data were collected through the gastric cancer information management system version 1.2 of Harbin Medical University Cancer Hospital (Copyright Registration No. 2013SR087424, official website). The data included the demographic characteristics of the patients, treatment methods, laboratory indicators, and pathological and histological results. Tumour staging was based on the 8th edition of the gastric cancer TNM staging system established by the American Joint Committee on Cancer/International Union Against Cancer (AJCC/UICC). Patient survival status was tracked every six months after discharge.

### Study endpoint

The primary endpoint of the study was the 5-year all-cause mortality rate.

### Follow-up

Patients with gastric cancer whose data were collected consecutively from January 2014 to December 2016 were included in the study. The follow-up period for each patient was standardised, starting from the date of discharge after surgical treatment and ending either when the 5-year follow-up period was completed or when the patient died during the follow-up period, whichever came first. Specifically, the follow-up initiation date for each patient was consistent with their individual discharge date after surgery, and the follow-up cut-off date was determined as follows: for patients who survived the entire follow-up period, the cut-off date was 5 years after their discharge date, and for patients who died during the follow-up period, the cut-off date was the date of death. The overall follow-up period for the study ranged from the discharge date of the first enrolled patient (January 2014) to the 5-year follow-up completion date of the last enrolled patient (December 2021), ensuring that all patients met the 5-year survival follow-up time span requirement as specified in the study endpoint.

### Data Processing (Missing Value Supplementation)

For missing value imputation in the dataset, the K-Nearest Neighbours Imputer (KNNImputer) from the scikit-learn library was adopted. Before imputation, the dataset was confirmed to be composed of numerical variables to meet the input requirements of KNNImputer. The key parameters of KNNImputer were set as follows: the number of neighbours (k value) was set to 5, and the default Euclidean distance was used as the distance measurement method to calculate the similarity between samples. The specific implementation process was as follows: first, the KNNImputer was initialised with n_neighbors = 5; then, the imputer was fitted on the original dataset to learn the distribution characteristics of the data; finally, the fitted imputer was used to transform the original dataset, completing the imputation of missing values. The imputed dataset was saved and used for subsequent model training and analysis. The specific code implementation of the imputation process is consistent with the standard operation of KNNImputer in scikit-learn, ensuring the reproducibility of the method.

### Feature selection

Initially, 40 clinical variables were included, and multicategory variables were processed with one-hot encoding. Key variables were selected through a recursive feature elimination (RFE) method with 10-fold cross-validation using random forests.

### Model development

In this study, an extreme gradient boosting (XGBoost) model was used to predict the 5-year all-cause mortality rate of patients with gastric cancer. The 2270 patients were randomly divided into a training set and a test set at a 7:3 ratio. The model was built based on the training set and evaluated on the test set, using various metrics to assess model performance. To further optimise the predictive performance of the XGBoost model, avoid overfitting, and improve model stability, this study adopted the Grid Search (GridSearchCV) method to systematically tune the key hyperparameters of the XGBoost model, determine the optimal hyperparameter combination, and ensure the prediction accuracy and reliability of the model.

A grid search was used for the hyperparameter tuning of the XGBoost model, with roc_auc as the core evaluation index, combined with 10-fold cross-validation (cv = 10), to determine the hyperparameter search range and screen the optimal combination. Finally, the optimal hyperparameters of the XGBoost model were obtained as follows: learning_rate = 0.1, max_depth = 2, and n_estimators = 50, and the remaining hyperparameters were set to default values. To comprehensively evaluate the predictive performance of the XGBoost model, this study further included four other common machine learning models for comparative analysis, namely, logistic regression (LR), decision tree (DT), K-nearest neighbours (KNN), and random forest (RF). These models were constructed using the same training set and test set as the XGBoost model, ensuring the fairness and rigour of the comparative study. All the models were evaluated using the same performance metrics to systematically compare the predictive advantages of the XGBoost model over other conventional machine learning models.

### Model interpretation and feature importance

The predictive model was explained using Shapley Additive Explanations (SHAP) values, and by ranking the importance of features, the main predictive factors affecting the survival of patients with gastric cancer were identified.

### Statistical analysis

All the statistical calculations were performed using Python version 3.9 and R language version 4.2.1. The performance of the machine learning models was assessed using metrics such as the area under the receiver operating characteristic curve (AUC), sensitivity, specificity, and F1 score. Differences between groups were analysed using the Pearson χ2 test or Fisher’s exact test. The Youden index was used to determine the optimal threshold for dividing patients into low-risk and high-risk groups. A P value of less than 0.05 was considered to indicate statistical significance.

## Results

### Patient characteristics

This study evaluated the 5-year survival status of 2270 patients with gastric cancer, of whom 71.3% (1619) were male and 28.7% (651) were female. The median age of the patients was 59 years (IQR: 52.00, 65.00). The primary origin site of gastric cancer was the pylorus (1573 cases, 69.3%), followed by the gastric body (336 cases, 14.8%), with the most common histological type being moderately to well-differentiated adenocarcinoma (1094 cases, 48.2%). By the end of the study, a total of 876 patients (38.5%) had died. The distribution of characteristics between the training and test sets was random and uniform (Table 1).

**Table 1:** Clinicopathological features of patients with gastric cancer.

*N*	Overall	Training set	Testing set	*P* value
	2270	1589	681	
sex (%)				
Male	1619 (71.3)	1141 (71.8)	478 (70.2)	0.466
Female	651 (28.7)	448 (28.2)	203 (29.8)	
age (median [IQR])	59.00 [52.00, 65.00]	59.00 [52.00, 65.00]	60.00 [53.00, 65.00]	0.039
pTNM (%)				
I	649 (28.6)	442 (27.8)	207 (30.4)	0.61
II	570 (25.1)	407 (25.6)	163 (23.9)	
III	945 (41.6)	664 (41.8)	281 (41.3)	
IV	106 (4.7)	76 (4.8)	30 (4.4)	
Borrmann (%)				
0	536 (23.6)	368 (23.2)	168 (24.7)	0.771
I	78 (3.4)	56 (3.5)	22 (3.2)	
II	506 (22.3)	356 (22.4)	150 (22.0)	
III	968 (42.6)	687 (43.2)	281 (41.3)	
IV	182 (8.0)	122 (7.7)	60 (8.8)	
Lauren (%)				
Intestinal	1221 (53.8)	853 (53.7)	368 (54.0)	0.584
Mixed	668 (29.4)	476 (30.0)	192 (28.2)	
Diffuse	381 (16.8)	260 (16.4)	121 (17.8)	
LVI (%)				
Negative	1146 (50.5)	805 (50.7)	341 (50.1)	0.833
Positive	1124 (49.5)	784 (49.3)	340 (49.9)	
PNI (%)				
Negative	721 (31.8)	477 (30.0)	244 (35.8)	0.007
Positive	1549 (68.2)	1112 (70.0)	437 (64.2)	
INF (%)				
INFa	813 (35.8)	585 (36.8)	228 (33.5)	0.099
INFb	826 (36.4)	556 (35.0)	270 (39.6)	
INFc	631 (27.8)	448 (28.2)	183 (26.9)	
HER2 (%)				
0	1227 (54.1)	854 (53.7)	373 (54.8)	0.852
1+	658 (29.0)	469 (29.5)	189 (27.8)	
2+	253 (11.1)	174 (11.0)	79 (11.6)	
3+	132 (5.8)	92 (5.8)	40 (5.9)	
chemotherapy (%)				
Yes	978 (43.1)	709 (44.6)	269 (39.5)	0.027
No	1292 (56.9)	880 (55.4)	412 (60.5)	
Histology (%)				
High-moderately	1094 (48.2)	758 (47.7)	336 (49.3)	0.297
poorly	507 (22.3)	366 (23.0)	141 (20.7)	
low adhesion	545 (24.0)	372 (23.4)	173 (25.4)	
mucinous	124 (5.5)	93 (5.9)	31 (4.6)	
Location (%)				
Low	1573 (69.3)	1107 (69.7)	466 (68.4)	0.483
Middle	336 (14.8)	233 (14.7)	103 (15.1)	
Upper	260 (11.5)	185 (11.6)	75 (11.0)	
Whole	101 (4.4)	64 (4.0)	37 (5.4)	
Tumor.size (median [IQR])	40.00 [25.00, 60.00]	40.00 [25.00, 60.00]	40.00 [25.00, 60.00]	0.129
WBC ×10⁹/L (median [IQR])	6.50 [5.37, 7.75]	6.49 [5.38, 7.74]	6.52 [5.36, 7.76]	0.866
LYM ×10⁹/L (median [IQR])	1.96 [1.60, 2.43]	1.97 [1.60, 2.46]	1.96 [1.62, 2.41]	0.613
NEU×10⁹/L (median [IQR])	3.71 [2.93, 4.69]	3.70 [2.93, 4.68]	3.74 [2.93, 4.72]	0.777
MONO×10⁹/L (median [IQR])	0.47 [0.37, 0.59]	0.47 [0.37, 0.59]	0.48 [0.38, 0.59]	0.232
EOD×10⁹/L (median [IQR])	0.12 [0.07, 0.20]	0.12 [0.07, 0.20]	0.12 [0.07, 0.21]	0.194
BASO×10⁹/L (median [IQR])	0.03 [0.02, 0.04]	0.03 [0.02, 0.04]	0.03 [0.02, 0.04]	0.439
RBC ×10¹²/L (median [IQR])	4.48 [4.07, 4.85]	4.47 [4.05, 4.85]	4.50 [4.10, 4.85]	0.286
HGB g/L (median [IQR])	136.74 [119.00, 149.30]	137.00 [118.20, 149.00]	136.00 [120.30, 150.00]	0.633
ALT U/L (median [IQR])	18.00 [14.00, 25.00]	18.00 [14.00, 25.00]	19.00 [14.00, 25.00]	0.923
AST U/L (median [IQR])	22.00 [18.00, 26.00]	21.00 [18.00, 26.00]	22.00 [18.00, 26.00]	0.994
YGGT U/L (median [IQR])	16.00 [11.00, 26.00]	16.00 [11.00, 26.00]	16.00 [11.00, 26.00]	0.808
LDH U/L (median [IQR])	157.00 [139.00, 176.00]	157.00 [139.00, 177.00]	157.00 [140.00, 175.00]	0.981
ALP U/L (median [IQR])	74.00 [62.00, 88.00]	74.00 [62.00, 88.00]	75.00 [62.00, 90.00]	0.338
PALB mg/L (median [IQR])	252.00 [202.00, 305.00]	252.00 [201.00, 305.62]	251.00 [206.00, 304.00]	0.695
GLU mmol/L (median [IQR])	5.10 [4.70, 5.70]	5.10 [4.70, 5.70]	5.10 [4.70, 5.70]	0.996
K mmol/L (median [IQR])	4.30 [4.03, 4.56]	4.30 [4.01, 4.60]	4.29 [4.04, 4.50]	0.396
NA. mmol/L (median [IQR])	142.00 [139.00, 144.00]	142.00 [139.00, 144.00]	142.00 [139.00, 144.00]	0.904
CL mmol/L (median [IQR])	104.00 [101.00, 106.00]	104.00 [101.00, 107.00]	104.00 [101.00, 106.00]	0.467
CA mmol/L (median [IQR])	2.24 [2.20, 2.40]	2.30 [2.20, 2.40]	2.20 [2.20, 2.40]	0.485
PHOS mmol/L (median [IQR])	1.14 [1.02, 1.26]	1.14 [1.01, 1.26]	1.14 [1.02, 1.25]	0.757
MG mmol/L (median [IQR])	0.97 [0.89, 1.04]	0.97 [0.90, 1.04]	0.96 [0.89, 1.03]	0.108
FBG g/L (median [IQR])	2.90 [2.44, 3.46]	2.90 [2.44, 3.46]	2.89 [2.45, 3.46]	0.889
CA199 U/mL (median [IQR])	10.55 [6.35, 20.25]	10.66 [6.26, 20.65]	10.16 [6.63, 18.11]	0.864
CEA ng/mL (median [IQR])	1.99 [1.20, 3.49]	2.02 [1.21, 3.58]	1.96 [1.19, 3.29]	0.487
CA724 U/mL (median [IQR])	2.73 [1.42, 6.29]	2.78 [1.42, 6.45]	2.66 [1.43, 5.85]	0.908
CA125 U/mL (median [IQR])	9.98 [7.42, 14.41]	10.07 [7.53, 14.68]	9.61 [7.21, 13.68]	0.022
BMI kg/m² (median [IQR])	22.68 [20.58, 24.97]	22.84 [20.68, 25.00]	22.43 [20.42, 24.84]	0.151

Note: Data are presented as n (%) for categorical variables and median [IQR] for continuous variables. Statistical analyses were performed using Pearson’s χ² test for categorical variables and Kruskal-Wallis test for continuous variables. *P* < 0.05 was considered statistically significant. Abbreviations: LVI, lymphovascular invasion; PNI, perineural invasion; INF, infiltration depth; HER2, human epidermal growth factor receptor 2; WBC, white blood cell; LYM, lymphocyte; NEU, neutrophil; MONO, monocyte; EOD, eosinophil; BASO, basophil; RBC, red blood cell; HGB, hemoglobin; ALT, alanine aminotransferase; AST, aspartate aminotransferase; GGT, gamma-glutamyl transferase; LDH, lactate dehydrogenase; ALP, alkaline phosphatase; PALB, prealbumin; GLU, glucose; K, potassium; NA, sodium; CL, chlorine; CA, calcium; PHOS, phosphorus; MG, magnesium; FBG, fibrinogen; CA199, carbohydrate antigen 199; CEA, carcinoembryonic antigen; CA724, carbohydrate antigen 724; CA125, carbohydrate antigen 125; BMI, body mass index.

### Data preprocessing and feature selection

**Fig. 1:**
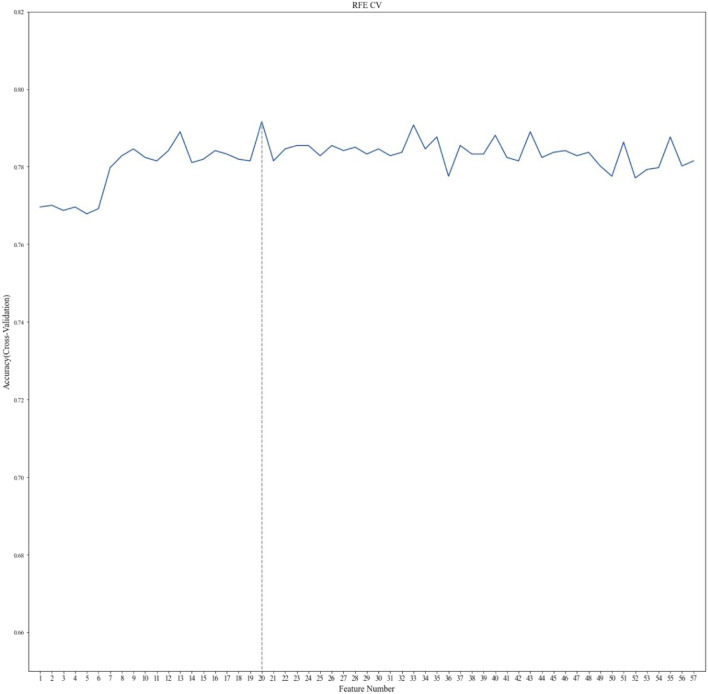
Results of feature screening by recursive feature elimination (RFE) with 10-fold cross-validation (10-fold CV). Initially, 40 clinical variables were included, and multi-category variables were processed by one-hot encoding to ensure valid algorithm input. Key variables were selected via RFE (with random forests as the base classifier) combined with 10-fold CV, which helped avoid overfitting and ensure the reliability of the selected features.

The KNNImputer method was applied to impute variables with a missing data rate of less than 30%, and non-ordinal multicategorical variables were processed using one-hot encoding. Through recursive feature elimination with a random forest (RFE-RF) feature selection method using 10-fold cross-validation, 20 key features were selected (Fig. 1), including age, pathological TNM staging, vascular cancer thrombus, neural invasion, tumour size, lymphocyte count, neutrophil count, red blood cell count, haemoglobin level, lactate dehydrogenase level, prealbumin level, serum phosphorus level, fibrinogen level, CA199, CEA, CA724, CA125, body mass index (BMI), Borrmann classification 0, and Borrmann classification IV (Table S1).

### Development and validation of machine learning models

Patients were randomly divided into a training set (1589 cases) and a test set (681 cases) at a ratio of 7:3. To comprehensively evaluate the predictive performance of different models, four common machine learning models (LR, DT, KNN, and RF) were constructed alongside the XGBoost model, with all the models trained on the training set and validated on the test set under the same evaluation criteria.

Among these models, the XGBoost model, constructed with 20 selected variables in the training set, exhibited the best overall comprehensive performance—although it did not achieve the highest value for every single indicator. Specifically, the performance metrics of the XGBoost model in the training set were as follows: an area under the curve (AUC) of 0.886, a sensitivity of 0.795, a specificity of 0.824, an accuracy of 0.811, a precision–recall curve of 0.831, a positive predictive value (PPV) of 0.748, a negative predictive value (NPV) of 0.853, a precision of 0.8, a recall of 0.804, and an F1 score of 0.802.

**Fig. 2:**
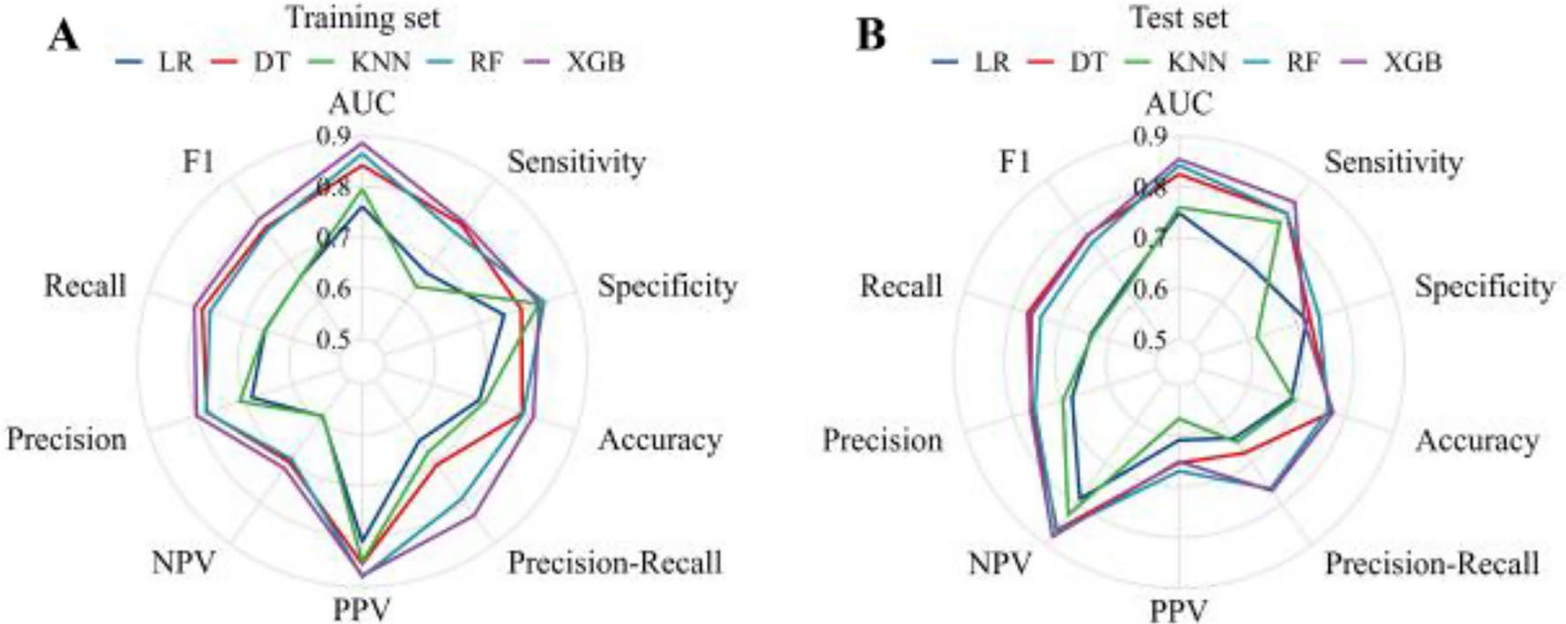
Performance comparison of five models (XGBoost, Decision Tree (DT), K-Nearest Neighbor (KNN), Random Forest (RF)) in the training and testing sets. The performance of each model was evaluated by ten indicators, including Area Under the Curve (AUC), Sensitivity, Specificity, Accuracy, Precision-Recall curve, Positive Predictive Value (PPV), Negative Predictive Value (NPV), Precision, Recall, and F1 score. This figure presents the performance results of the five models across all indicators in both the training and testing sets, facilitating the comparison of their predictive capabilities.

In the test set, the performance metrics of the XGBoost model were an AUC of 0.855, a sensitivity of 0.844, a specificity of 0.715, an accuracy of 0.775, a precision–recall curve of 0.768, a PPV of 0.713, an NPV of 0.813, a precision of 0.763, a recall of 0.761, and an F1 score of 0.762. Compared with the other four models (LR, DT, KNN, and RF), the XGBoost model showed superior comprehensive performance in terms of overall predictive accuracy, stability, and generalisation ability, fully demonstrating its ability to predict the related outcomes of patients with gastric cancer (Fig. 2, Table S2). A calibration curve was generated to evaluate the calibration of the predictive model, and the detailed calibration curve is shown in Figure S1. In addition, the XGBoost model showed the best performance in both the training set and the test set.

### Visualization of feature importance

**Fig. 3:**
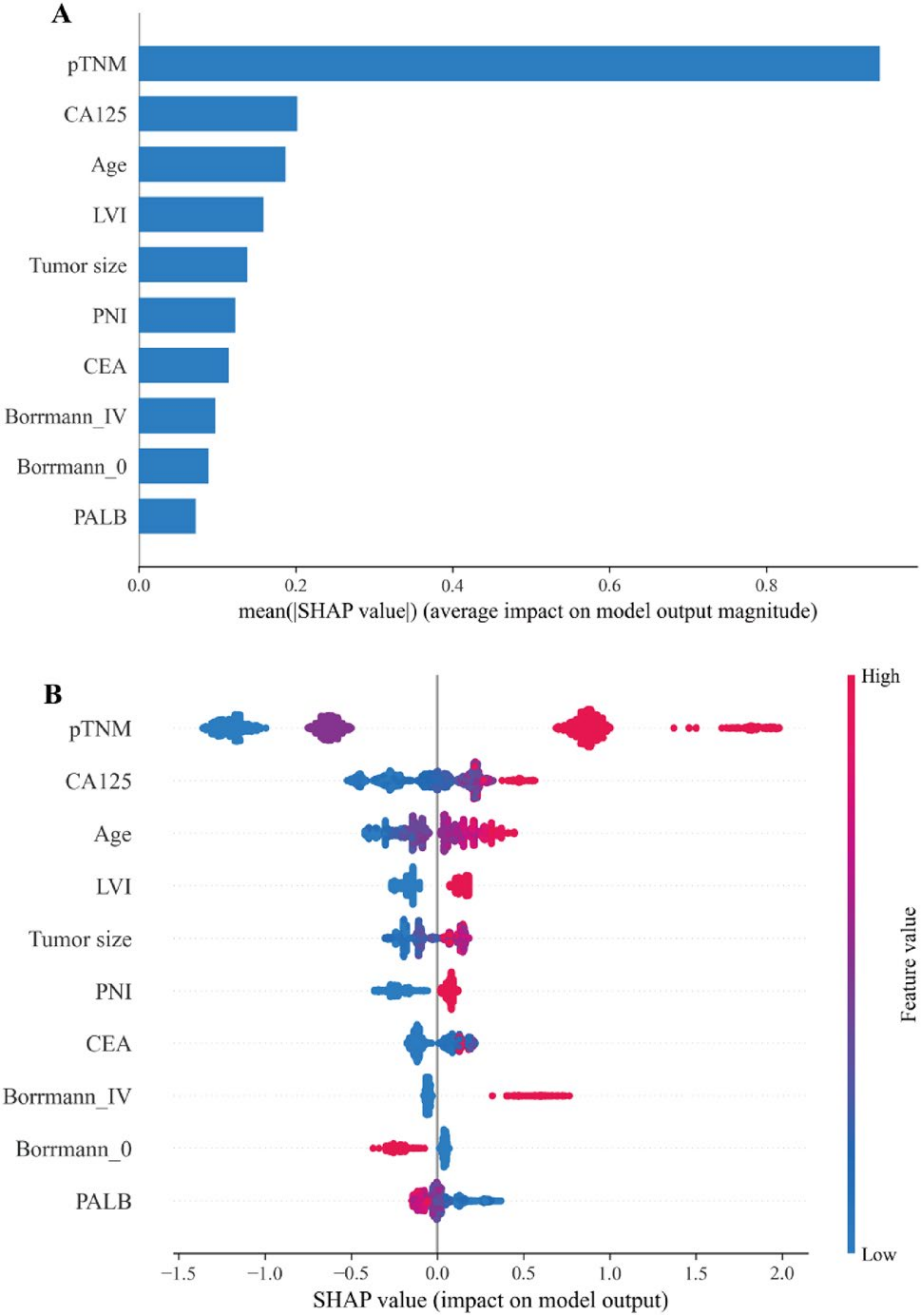
Interpretation of the prognostic model. SHAP (SHapley Additive exPlanations) values were used to rank the importance of the top 10 clinical variables based on their mean values, which reveals the key features most closely associated with the survival risk of gastric cancer patients (Fig. 3A). After model optimization, the top 10 prognostic risk factors were further ranked by their importance; in this visualization, higher feature values (marked in red) indicate an increased risk of death in patients (Fig. 3B).The top 10 variables (pTNM: pathological TNM staging, Age: patient age, CA125: carbohydrate antigen 125, CEA: carcinoembryonic antigen, LVI: lymphovascular invasion, Tumor size: tumor diameter, CA199: carbohydrate antigen 199, PNI: prognostic nutritional index, HGB: hemoglobin, PALB: palbociclib).

The use of SHAP values to rank the importance of the top 10 variables by mean revealed the features most related to patient survival risk (Fig. 3A). After the model was optimised, the top 10 risk factors affecting prognosis were ranked by importance, with higher feature values (in red) indicating an increased risk of patient death (Fig. 3B). To make the feature importance analysis more intuitive and verifiable, a detailed quantitative table (Table S3) has been added, which presents the key features along with their specific mean absolute SHAP values and full importance rankings.

### Explanation of personalized prediction

**Fig. 4:**
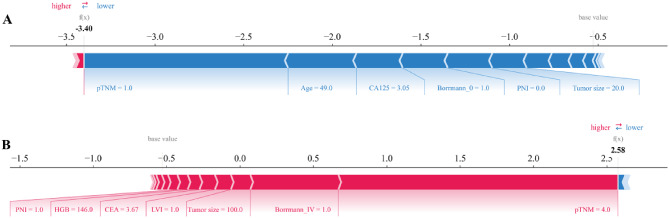
Interpretation of the model prediction results using two patient samples. (**A**) Survival case; (**B**) Non-survival case. Two patient cases were selected to illustrate the explanatory power of the prognostic model: one was a Stage I gastric cancer patient with negative perineural invasion (PNI), indicating long-term survival; the other was a Stage IV patient with positive PNI, who died within 5 years. Arrows in the figure indicate the direction of each variable’s impact on the model’s prediction outcome, where red arrows represent an increased risk of death and blue arrows represent a decreased risk of death.

Two patient cases are presented to illustrate the explanatory power of the model: one is a Stage I patient with negative perineural invasion (PNI), indicating long-term survival; the other is a Stage IV patient with positive PNI, who died within 5 years. The arrows indicate the direction of each variable’s impact on the prediction outcome, with red and blue arrows representing an increase and decrease in the risk of death, respectively. The SHAP values and predicted scores, which were calculated by integrating the effects of all the variables, reflected lower SHAP values (-3.4) and predicted scores (0.032273) for surviving patients and higher SHAP values (2.58) and predicted scores (0.929268) for deceased patients (Fig. 4).

### Risk stratification

The optimal threshold for risk stratification was determined using the ROC curve and the Youden index for the test set. A Youden index of 0.6189 and a corresponding cut-off value of 0.42731 were used to classify patients into high-risk and low-risk groups.

**Fig. 5:**
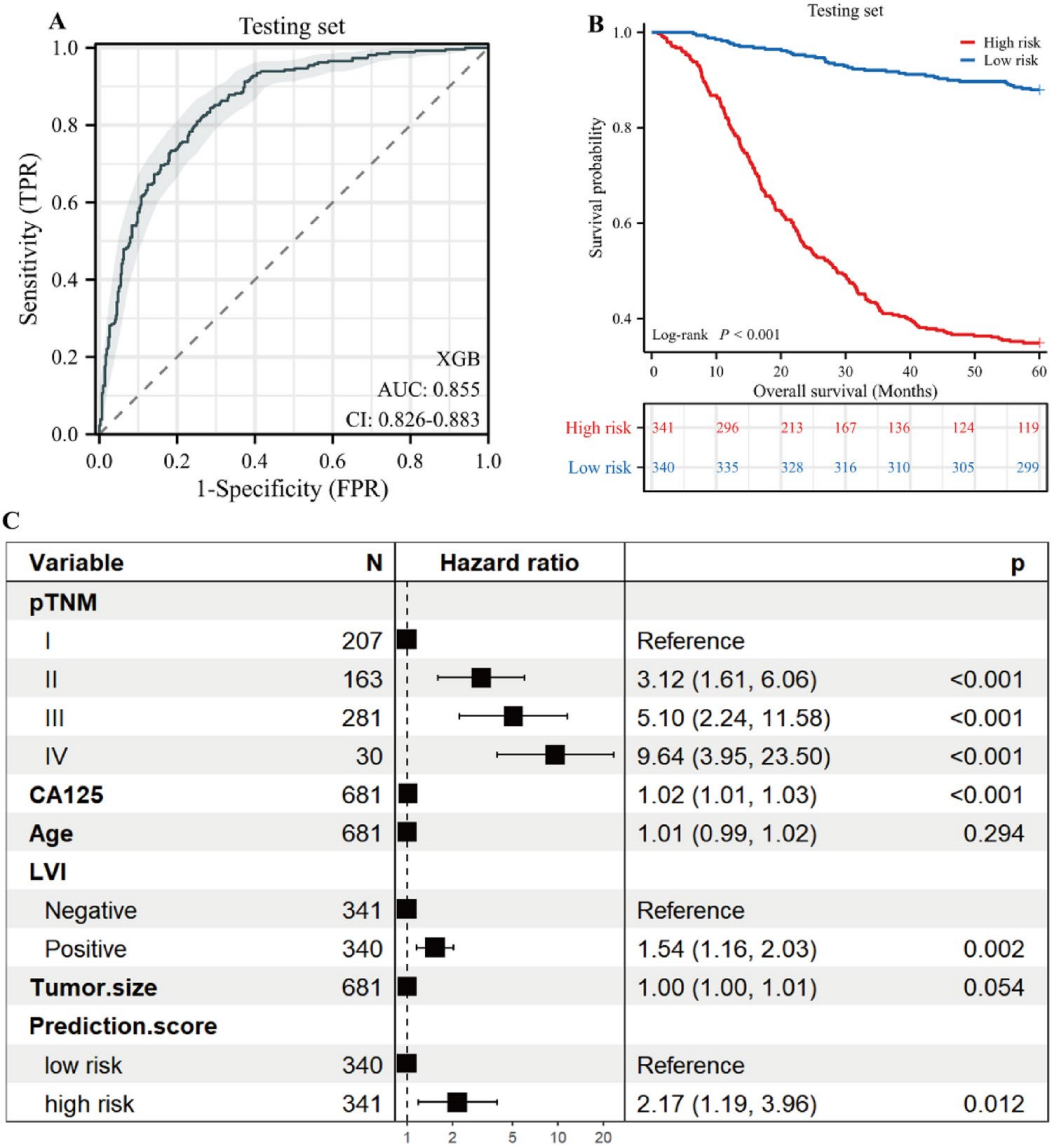
(**A**) ROC curve of the XGBoost model in the test set; (**B**) Kaplan-Meier survival curve for patients with low and high risk scores in the test set; (**C**) Multivariable Cox regression analysis for 5-year all-cause death prediction in the test set. The optimal risk stratification threshold was determined by the Youden index of the test set, which was used to divide patients in the test set into high-risk and low-risk groups. Survival analysis showed significantly worse prognosis in the high-risk group of the test set. Multivariate Cox regression analysis incorporating the model’s prediction score and key clinical features in the test set confirmed that the prediction score is an independent prognostic risk factor.

Survival analysis revealed that the prognosis of patients in the high-risk group was significantly worse than that in the low-risk group (*P* < 0.001). When the prediction score and the five most important features (pTNM stage, CA125 concentration, age, vascular cancer thrombus, and tumour size) were incorporated into a multivariate analysis, the prediction score was an independent risk factor for patient prognosis (hazard ratio [HR]: 2.17; 95% confidence interval [CI]: 1.19–3.96; *P* = 0.012) (Fig. 5).

## Discussion

This study successfully developed a prognostic prediction model for patients with gastric cancer based on the XGBoost algorithm and key clinical features such as pTNM staging, CA125 level, age, vascular tumour thrombus, and tumour size. Our model demonstrated good predictive ability in the test set, with a high AUC, sensitivity, specificity, and accuracy among its evaluation metrics. These findings indicate that the use of the XGBoost algorithm for predicting the prognosis of gastric cancer is feasible and effective, which is consistent with the successful application of this algorithm in the prognostic prediction of various types of cancer in recent years^18–22^.

Notably, the most important predictive variables identified in our model were pTNM staging and CA125 level, highlighting the significant role of tumour stage and biomarkers in predicting the prognosis of patients with gastric cancer.

The pTNM staging system is an internationally recognised and irreplaceable tool for predicting the prognosis of patients with tumours^23^. Similarly, CA125, a commonly used tumour marker in clinical practice, has significant guiding value for clinicians in assessing the prognosis of patients with gastric cancer^24^. As a core prognostic factor identified in our study, CA125 is closely associated with the 5-year survival of patients with gastric cancer, and its prognostic role is linked to key clinical and pathological processes, especially tumour invasion and metastasis. Mechanistically, CA125, a classic tumour-associated antigen, affects the prognosis of gastric cancer mainly by promoting tumour invasion and peritoneal metastasis. Clinical studies have shown that serum CA125 levels are significantly higher in patients with gastric cancer who have deep tumour invasion and peritoneal metastasis, and a CA125 concentration ≥ 14.4 KU/L is an independent risk factor for poor survival. This relationship exists because CA125 enhances the adhesion of gastric cancer cells to the peritoneal surface and extracellular matrix by regulating β1-integrin and CD44H expression, thereby accelerating peritoneal metastasis. Additionally, an elevated CA125 level is positively correlated with an advanced TNM staging and poor tumour differentiation, indirectly reflecting tumour aggressiveness and affecting long-term survival. Collectively, CA125 affects the 5-year survival of patients with gastric cancer mainly by promoting tumour invasion and metastasis and by correlating with adverse pathological features, confirming its value as a core prognostic factor^25,26^.

The comprehensive importance of these variables was further confirmed by the construction of the gastric cancer prognosis prediction model in this study. Furthermore, the effects of factors such as age, vascular tumour thrombus, and tumour size on the prognosis of patients with gastric cancer were revealed in this study. The findings of many scholars are consistent with those of this study^27–29^, further confirming the necessity of using multidimensional clinical features in the construction of prognostic models. The presence of vascular tumour thrombi should also be given adequate attention, as this factor is directly linked to tumour invasion and spread and is among the key factors guiding clinicians to make reasonable treatment plans and prognostic assessments for patients^30^.

To increase the clinical value of the study, the specific clinical application scenarios of the XGBoost predictive model were refined in combination with the clinical practice for gastric cancer. The model can be effectively applied in three core clinical scenarios: preoperative prognostic assessment, the formulation of postoperative follow-up strategies, and individualised intervention for high-risk patients. Specifically, the preoperative application of the model helps clinicians evaluate the disease progression potential and prognosis of patients, providing a scientific basis for formulating personalised surgical plans. In the postoperative period, the model can stratify patients into high-risk and low-risk groups, guiding clinicians to develop targeted follow-up schedules (e.g., increasing the frequency of re-examinations for high-risk patients to facilitate the early detection of tumour recurrence). For high-risk patients identified by the model, timely individualised intervention measures (such as adjuvant chemotherapy) can be implemented to optimise treatment effects and improve patient prognosis.

While the prognostic model developed in this study demonstrates good prognostic assessment performance, some limitations are unavoidable. First, as this was a retrospective study, the comprehensiveness of data collection may be limited; in subsequent research, we will conduct multicentre prospective studies to avoid potential biases caused by retrospective data and improve the reliability of the research results. Second, although our model performed well in the internal test set, the generalisability of the prognostic model across different populations and health care settings cannot be assessed; thus, we plan to perform external validation in multiple independent cohorts to verify the model’s applicability in different clinical scenarios. Furthermore, the emergence of numerous molecular markers^31,32^ and genetic information^33,34^ could further improve prediction accuracy, but our model’s reliance solely on traditional clinical features may present certain limitations. In follow-up research, we will integrate molecular biological characteristics and genomic data into the model to optimise its prediction performance.

With the pressing demand for personalised diagnosis and treatment, the integration of molecular biological characteristics and genomic data into prognostic models has become a mainstream trend. This more comprehensive information can provide deeper insights into the prognostic assessment of gastric cancer. Therefore, integrating the aforementioned molecular information into existing models to further enhance model accuracy and clarify its correlation with clinical behaviour is a very promising direction for future work.

## Conclusion

This study successfully established a predictive model for the five-year survival rate of patients with gastric cancer by integrating the XGBoost algorithm with key clinical indicators. The results of this study indicate that the constructed model can provide a reliable reference for the prognostic assessment of patients with gastric cancer and preliminarily demonstrate the application potential of machine learning-based approaches in aiding personalised treatment decision-making and the optimisation of cancer prognostic assessment methods.

## Electronic Supplementary Material

Below is the link to the electronic supplementary material.

Supplementary Material 1

## Data Availability

The data underlying this article cannot be shared publicly due to privacy concerns regarding the individuals involved in the study. The data will be shared on reasonable request to the corresponding author.
