# Supplementary material for: XGBoost-based model for predicting five-year survival in gastric cancer using clinical indicators
Source: Sci Rep. 2026 Apr 25;16:19209. doi: 10.1038/s41598-026-50043-x (PMC13284183; doi:10.1038/s41598-026-50043-x)
Supplement: Supplementary file 1 — Supplementary Material 1 [file 41598_2026_50043_MOESM1_ESM.pdf]

Supplementary File 1: Structured Questionnaire for Clinicodemographic and Clinical Data Collection

Study Title: Evaluation of Screening Tests and Microbial Profile of Asymptomatic Bacteriuria Among Pregnant Women at a Tertiary Care Hospital in India

Participant Identification

1. Participant ID: __________
2. Date of enrolment: ___ / ___ / ______
3. OP/IP Number: __________

Demographic Details

1. Maternal Age (years): __________
2. Residence:
   ☐ Urban ☐ Rural
3. Socioeconomic Status (SES):

   (Based on income, occupation, and living conditions)

   ☐ Low

   ☐ Middle

   ☐ High

Obstetric Profile

1. Gestational Age (weeks): __________
2. Gravida (Number of pregnancies): __________
3. Parity (Number of viable births): __________
4. Type of Previous Delivery (if applicable):

   ☐ Normal Vaginal Delivery

   ☐ Caesarean Section

   ☐ Instrumental Delivery

   ☐ Not Applicable

Obstetric History

(Previous pregnancies, if any)

1. History of PROM (Premature Rupture of Membranes):

   ☐ Yes ☐ No

2. History of Preterm Delivery:

   ☐ Yes ☐ No

3. History of Low Birth Weight Baby (<2.5 kg):

   ☐ Yes ☐ No

4. Any other complications (specify):

Medical and Clinical History

1. History of Catheterization:

   ☐ Yes ☐ No

2. Previous History of Urinary Tract Infection (UTI):

   ☐ Yes ☐ No

3. History of Other Infections (specify):

   ☐ Yes ☐ No

   If yes, specify: ______________________

Comorbidities

(As per documented clinical diagnosis or medical records)

1. Anaemia:

   ☐ Yes ☐ No

2. Diabetes Mellitus:

   ☐ Yes ☐ No

3. Hypertension:

   ☐ Yes ☐ No

4. Renal Disorders:

   ☐ Yes ☐ No

5. Any other comorbidity (specify):

Blood Group

1. ABO Blood Group:

   ☐ A ☐ B ☐ AB ☐ O

2. Rh Factor:

   ☐ Positive ☐ Negative

Consent

I confirm that I have been informed about the study and consent to participate.

Signature/Thumb impression:

Date: ___ / ___ / ______
